# Cerebrovascular accidents associated with hip fractures: morbidity and mortality—5-year survival

**DOI:** 10.1186/s13018-018-0867-1

**Published:** 2018-06-28

**Authors:** Ran Atzmon, Zachary T. Sharfman, Noa Efrati, Noam Shohat, Yaron Brin, Iftach Hetsroni, Meir Nyska, Ezequiel Palmanovich

**Affiliations:** 1 Department of Orthopaedic Surgery, Assuta Medical Center, Affiliated with the Faculty of Health and Science and Ben Gurion University, Ashdod, Israel; 2 Montefiore Department of Orthopaedic Surgery, Bronx, New York, USA; 3 Department of Orthopaedic Surgery, Meir Hospital Sapir Medical Center, Affiliated with the Tel Aviv University Sackler Faculty of Medicine, Kfar Saba, Israel; 4 Department of Orthopaedic Surgery, Assaf Harofeh Medical Center, Affiliated with the Tel Aviv University Sackler Faculty of Medicine, Zerifin, Israel

**Keywords:** Stroke, Hip fracture, Cerebrovascular accident, Mortality

## Abstract

**Background:**

Hip fractures are associated with increased cerebrovascular accidents (CVAs) in the first postoperative year. Long-term follow-up for CVA and mortality after hip fracture is lacking. The purpose of this study was to identify risk factors for CVA and follow mortality in hip fractures in a cohort with greater than 2 years follow-up.

**Methods:**

We compared past medical history of patients with hip fractures to long-term survival and the occurrence of CVA. Past medical history, surgical intervention, CVA occurrence, and death were queried from the electronic medical recorder system. Level of significance was set at *p* < 0.05 with 95% confidence interval.

**Results:**

Two thousand one hundred ninety-five patients met inclusion criteria. Mean follow-up was 5 years. One hundred ten (5.01%) patients were diagnosed with post-fracture CVA. Forty-one patients had CVA in the first year and 55 patients had CVA between 1 to 5 years after surgery. Among the potential risk factors, hypertension (HTN), atrial fibrillation (AF), and diabetes mellitus (DM) had the highest odds ratio for CVA (OR = 1.885, *p* value = 0.005; OR = 1.79, *p* value = 0.012; OR = 1.66, *p* value = 0.012). The median survival time in patients with CVA was 51.12 ± 3.76 months compared to 59.60 ± 0.93 months in patients without CVA (*p* = 0.033).

**Conclusions:**

HTN, AF, and DM are significant risk factors for the occurrence of CVA after hip fracture. The majority of CVAs occur between the first and fifth year postoperatively, and CVA is a negative prognostic factor for postoperative survival.

## Background

Hip fractures are common in the elderly population and have tremendous implications for patient health and life expectancy. It is estimated that more than 250,000 people in the USA experience a hip fracture each year [1]. Risk factors predisposing to hip fractures include osteoporosis, low patient mobility, low physical fitness, pharmacological factors, low socioeconomic status, and co-morbidities such as patients after cerebrovascular accidents (CVAs) [2, 3]. It is reported that between 11 and 23% of patients die within 6 months after hip fracture and between 22 and 37% die within the first year [4–7].

Hip fracture and CVA are reportedly inter-related. Hip fracture has been identified as a significant risk factor for CVA, while CVA has also been reported to significantly increase the risk of a future hip fracture [8, 9]. The importance of identifying risk factors for CVA after hip fracture cannot be understated, as the negative implications to a patient’s health, the increased risk of post-fracture death, and the economic and societal burdens are cumbersome. Currently, however, information about the association of hip fractures and CVA is limited. Studies exploring risk factors for CVA after hip fractures are generally limited by a short follow-up period of 1 year after the initial insult. The purpose of this study was to identify risk factors for CVA and follow mortality in hip fractures in a cohort with greater than 2 years follow-up. We hypothesized that (1) CVA after hip fracture has increase mortality rate, (2) patients with increase pre-operative morbidities should have increased incidence of CVA, (3) the majority of CVA events would occur in the first-year post fracture, and (4) hip fracture patients with CVA would have decreased survival compared to hip fracture patients without CVA.

## Methods

The local institutional review board approved this study. The collected data were retrospectively reviewed for all patients who presented to a level 2 trauma center with hip fractures between 2003 and 2014. Minimum follow-up was 2 years. Inclusion criteria were as follows: (1) patients who were diagnosed with femoral neck fractures according to the International Classification of Diseases, 9th Revision, Clinical Modification codes 820–821 and who were admitted to the department of orthopedic surgery and underwent surgery and (2) minimum 2 years follow-up. Exclusion criteria were as follows: (1) lack of medical record documentation, (2) hip fractures managed non-operatively, (3) less than 2 years follow-up, and (4) pathological fractures related to cancer. All patients received low-molecular-weight heparin (Enoxaparin), 40 mg intramuscular for 35 days from the time of admission for venous thromboembolic disease prophylaxis.

Data collected included sex, age, and fracture type—(1) extracapsular fracture and (2) intracapsular fracture. The time from admission to operation, time from operation to discharge, postoperative weight bearing protocol—(1) non-weight bearing (NWB), (2) partial weight bearing (PWB), (3) weight bearing as tolerated (WBAT), and (4) full weight bearing (FWB)—were recorded. The rehabilitation protocol—(1) home rehabilitation and (2) designated rehabilitation institutions—and anticoagulant use were recorded in addition to past medical history—(1) hypertension (HTN), (2) diabetes mellitus (DM), (3) hypercholesterolemia (HCL), (4) atrial fibrillation (AF), (5) ischemic heart disease (IHD), (6) congestive heart failure (CHF), (7) chronic renal failure (CRF), (8) deep vein thrombosis (DVT), and (9) pulmonary embolism (PE).

### Statistical analysis

Statistical analysis was conducted using IBM SPSS 21. Means and standard deviations were used to present continuous variables. Kaplan-Meier estimators were used to analyze 5-year survivorship. Chi-squared tests and *T* tests were used for categorical values and continuous variables, respectively. Logistic regression model was used to test inter-relationships between the potential risk factors and the occurrence of postoperative CVA. Level of significance was set at *p* < 0.05 with 95% confidence interval.

## Results

**Fig. 1:**
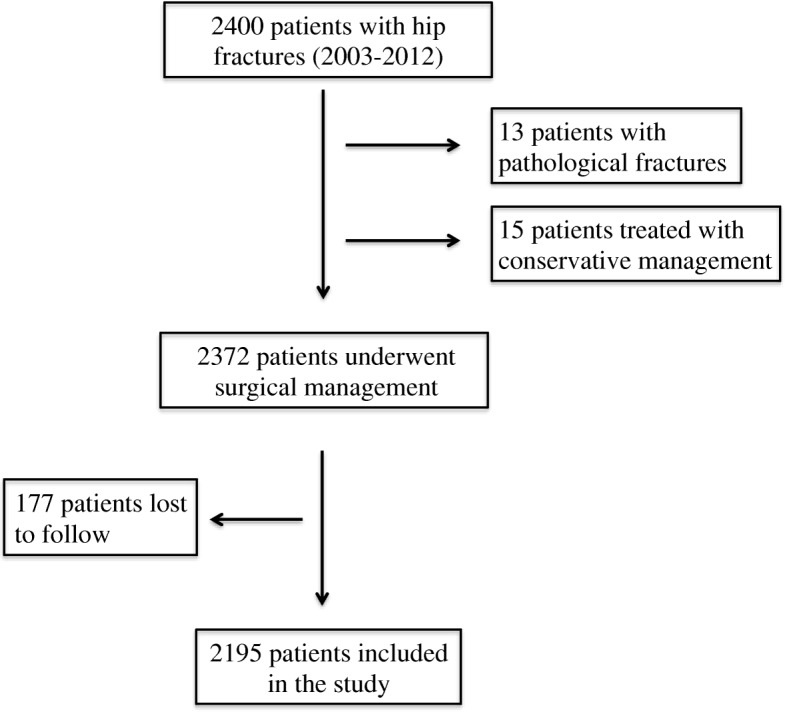
Inclusion flow chart

Two thousand four hundred patients were treated for hip fractures between 2003 and 2014. Two thousand one hundred ninety-five patients met the criteria for inclusion (Fig. 1). The mean age of the cohort was 79.3 years. There were 1474 (67.15%) women. Of the 2195 hip fracture cases included in the study, 385 patients (17.5%) were diagnosed with CVA. Two hundred seventy-five (12.5%) patients had CVA prior to fracturing their hip. Eighty-three (3.8%) patients had post fracture CVA (without previous history of CVA), and 27 (1.2%) patients had both pre and post fracture CVAs. In total, 110 (5%) patients sustained post fracture CVA (Table 1). Of the 110 patients who had post fracture CVA, 97 (93%) patients had CVA during the first 5 years post fracture. Nineteen had CVA during the 45 days after the fracture, with a mean time to CVA of 5.26 days. All patients who sustained CVAs during the first 45 days were receiving prophylactic Enoxaparin. Only one patient, who had already received prophylactic Enoxaparin, sustained a CVA while awaiting surgery. Forty-one patients had CVAs in the first year post fracture whereas 55 patients had CVAs in years 1 through 5. The timing of CVA in relation to hip fracture events is reported in Table 2. We analyzed the incidence of post fracture CVA by sex and age. There was no significant difference between the age groups or the sexes in terms of post fracture CVA (Table 3).

**Table 1:** CVA prevalence

Patient cohorts	Cohort number (*n*)	Percentage of total
No CVA	1810	82.4
Pre fracture CVA	275	12.5
Post fracture CVA (No prior history of CVA)	83	3.8
Pre and post fracture CVA	27	1.2
Post fracture CVA total	110	5

**Table 2:** Timing of CVA in relation to the hip fracture event

Time	*N*	Percentage
While awaiting surgery	1	0.9
POD 1–45 months	19	17.3
POD 45–6 months	8	7.3
6 months–1 year	13	11.8
1 year–5 years	55	50
> 5 years	7	6.3
Unknown POD	7	6.3
Total	110	100

**Table 3:** Age and sex distribution of post fracture CVA

Age group	Male	Female	Total
< 65	1	3	4 (3.64%)
65–74	7	12	19 (17.27%)
> 75	23	64	87 (79.09%)
Total	31	79	110 (100%)

*No significant relationships (*p* value 0.652)

We tested the relationship between potential risk factors and post fracture CVA. Patients with HTN had the highest odds ratio of CVA (OR = 1.885, *p* value = 0.005) followed by AF and DM (OR = 1.79, *p* value = 0.012 and OR = 1.66, *p* value 0.012, respectively) (Table 4). Other factors such as hypercholesterolemia, prior Coumadin use, ischemic heart disease, congestive heart failure, chronic renal failure, rehabilitation facilities, and weight bearing protocols did not significantly affect odds ratio of post fracture CVA. The co-existence of HTN, AF, and DM in a patient corresponded with a 2.5–3 times increased risk of CVA after a hip fracture.

**Table 4:** Odds ratio for developing CVA after hip fracture in relation to the potential risk factors

	Post fracture CVA status		
	+	−	*p* value
Age	80.7 ± 7.6	79.2 ± 12.3	0.204
Hypertension	76.4%	63%	0.005*
Diabetes mellitus	37.3%	26.4%	0.012*
Atrial fibrillation	22.7%	14.1%	0.012*
Hypercholesterolemia	31.2%	25.9%	0.218
Coumadin	5.5%	6.3%	0.709
Ischemic heart disease	25.5%	27.7%	0.602
Congestive heart failure	12.7%	12%	0.818
Chronic renal failure	7.3%	10.8%	0.437
Rehabilitation	33.6%	33.4%	0.964
Weight bearing	8.2%	9.3%	0.413
Sex (M/F)	M 71.8%, F 28.2%	M 67%, F 33%	0.295

**Fig. 2:**
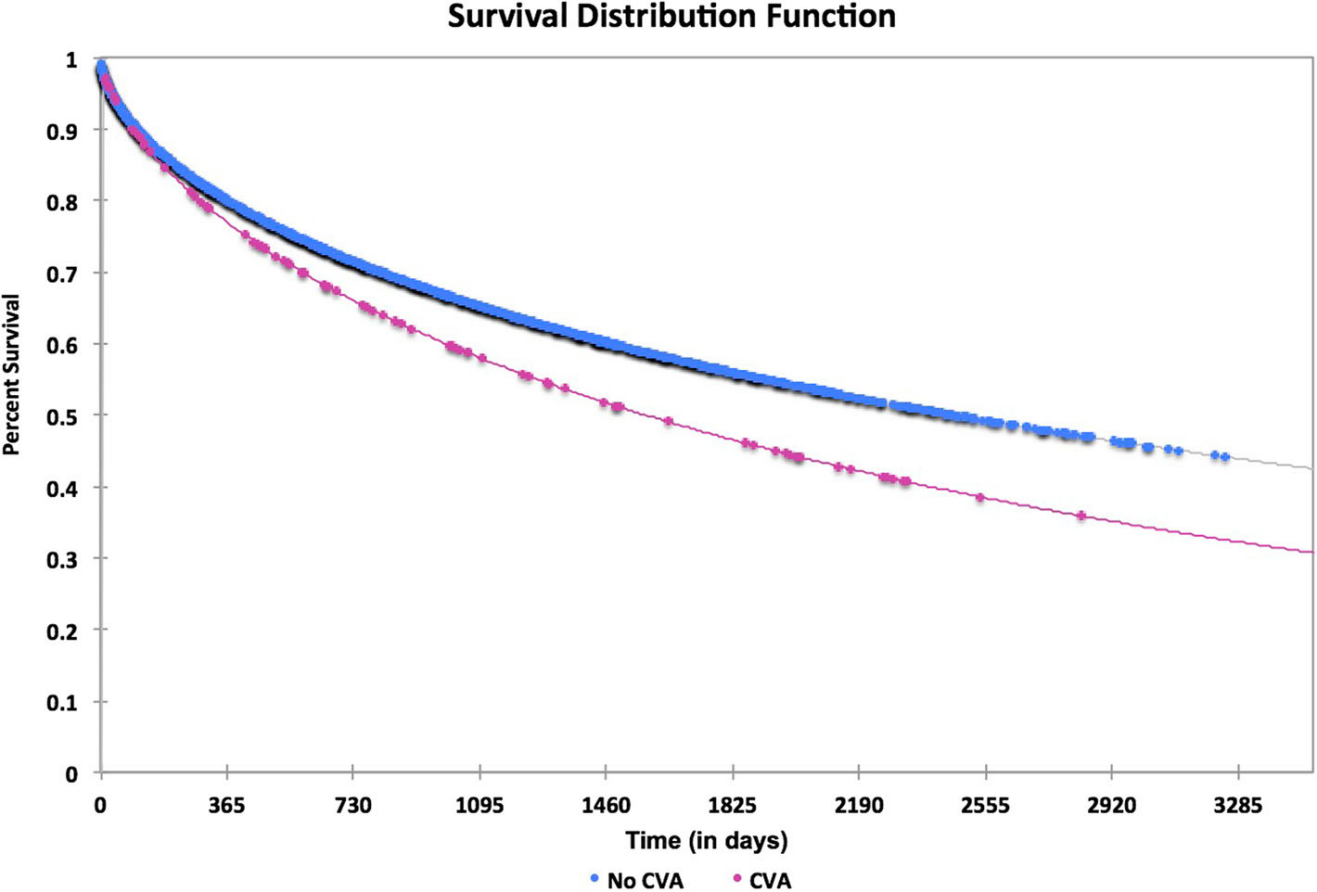
Kaplan-Meier survival distribution function

**Fig. 3:**
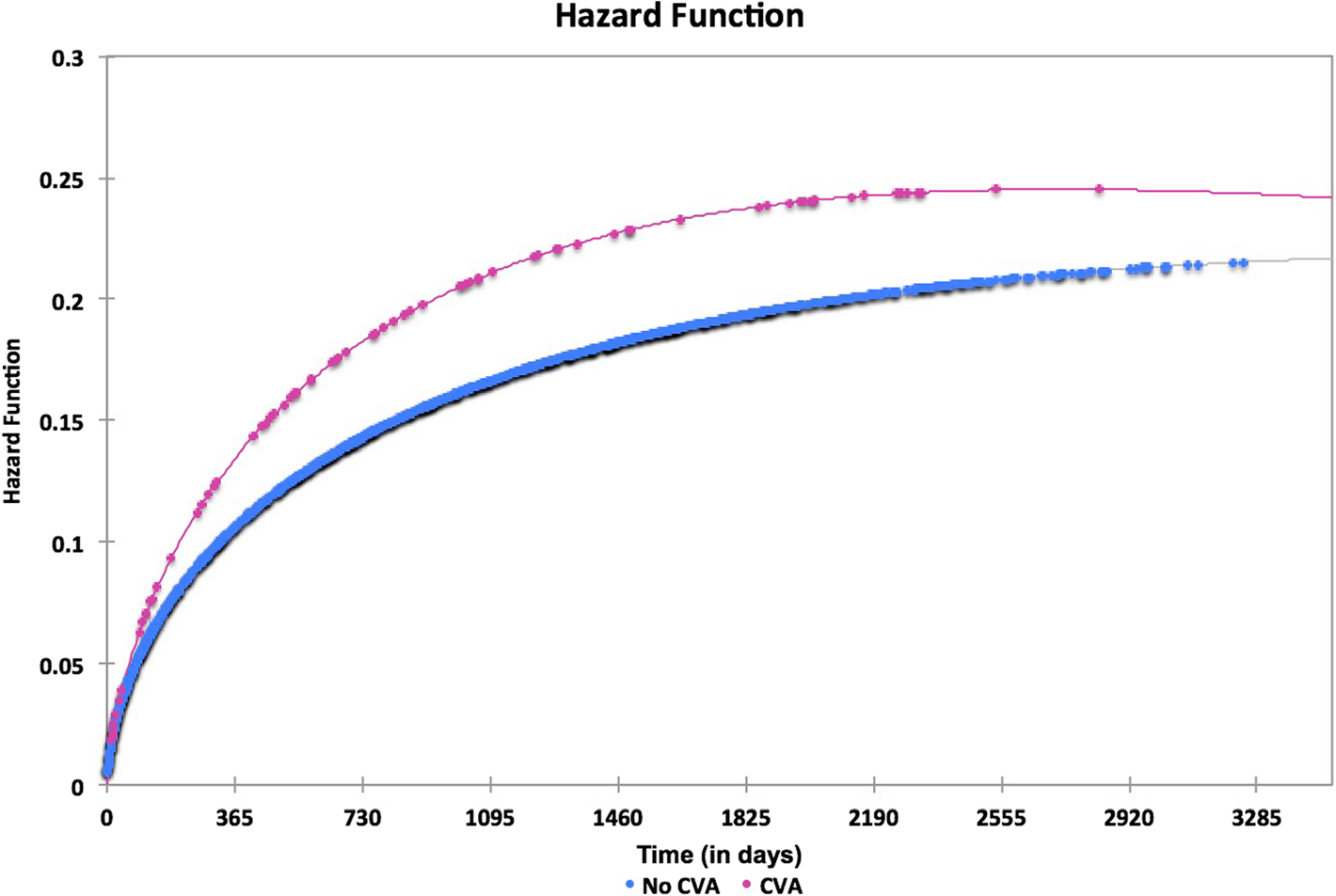
Kaplan-Meier survival hazard function

We analyzed the number of risk factors with respect to age and gender in all hip fracture patients (Table 4). Females under 65 had significantly more comorbidities on than their male counterparts (males 0.87 ± 1.37 females 1.34 ± 1.79, *p* = .032) whereas males older than 75 had significantly more comorbidities than their female counterparts (males 2.18 ± 1.53 females 2.00 ± 1.47, *p* = .025). Of the 110 patients with CVA, 77(70%) died within 5 years, compared to 1224 of the 2085 patients without CVA (58.71%). The median survival time in patients without CVA was 59.60 ± 0.93 months compared to 51.12 ± 3.76 months in patients with post fracture CVA (*p* = 0.033). The Kaplan-Meier survival distribution function and hazard function are displayed in Figs. 2 and 3 respectively.

There was no statistical difference between type of the fracture whether intracapsular or extracapsular and CVA (*p* = 0.098), though there were more patients with extracapsular fracture and CVA.

There was no statistical difference between the postoperative physiotherapy protocol (FWB, PWB, and NWB) in CVA incidence (*p* = 0.718).

## Discussion

The major finding of this study reports that patients with HTN, AF, and DM had increased risk for post hip fracture CVA and decreased survival. CVA is a leading cause of morbidity and mortality worldwide, and the association between hip fracture and CVA has been well documented [10, 11]. However, long-term follow-up, greater than 1 year, in the post hip fracture population, is limited. Moreover, the association between specific vascular-associated comorbidities and the occurrence of post hip fracture CVA was not previously well established.

Pathophysiologic mechanisms of CVA involve systemic migration of emboli to the brain through inter-cardiac or extra-cardiac origins. Sources of these emboli include deep vein thrombosis, fat emboli, septic emboli, and thromboembolism especially in the presence of atrial fibrillation. Edelsber et al. [12] specifically demonstrated the relationship between venous thromboembolism following major orthopedic surgery. Wilson et al. [13] and Adunsky et al. [14] contributed to the understanding of increased coagulation following orthopedic trauma and increased mortality in patients with AF following hip fractures, respectively. Invasive management is the standard treatment regimen for most hip fractures [15]. Surgeons should consider the pro-coagulative state induced by surgery, especially in patients with pre-existing vascular comorbidities that predisposes this at-risk population to post fracture CVA.

DM in itself is a risk factor for falls that may result in hip fracture [16]. Additionally, diabetes mellitus is a risk factor for CVA as supported by the findings of this study. This emphasizes the complex inter-relationship between hip fracture, CVA, and diabetes, as previously suggested by Donnan et al. [17].

The metabolic, physiologic, and physical insult of hip surgery is well known during the immediate postoperative period [18]. For example, Aicale et al. showed high prevalence of hyponatremia in the elderly patients with hip fractures, which was approximately 20% [19]. Additionally, neuromuscular, visual impairment, dementia, low bone density, alcohol intake, and smoking were found to be associated to fracture incidence and outcome [20]. Furthermore, the fixation technique, such as minimal invasive surgery, may also influence the postoperative complications, probably due to surgery time, blood loss, and length hospital stay [21]. And finally, one should take into account other factors such as seasonal incidence which was also shown to correlate with hip fracture as demonstrated by Douglas et. al. [22]

Special care is taken to balance risk factors in this crucial time to decrease postoperative complications. Following the rehabilitation period, when a patient reaches a plateau phase in recovery, regularly scheduled follow-up and close monitoring of risk factors is often neglected. One year after a hip fracture, patients and physicians may feel a false sense of security as the immediate hazards of surgery fade. This study reports that CVA is common even after 1 year from the original insult, and in accordance with these findings, risk factors predisposing to CVA must be continuously and closely monitored even after 1 year. In patients with DM, HTN, or AF and in patients with multiple of these comorbidities, long-term monitoring and management of their conditions is of even greater importance.

Older women comprise the majority of hip fracture patients [1]. Yet, risk of CVA is higher in men [23]. In this study, there was an inverse correlation between comorbidity burden and age in males and females. Older men had higher comorbidity burden relative to women but experienced less CVA. This demonstrates that female gender is a strong predictor of CVA after hip fracture regardless of comorbidity burden. Part of this effect may be due to increased cardiovascular risk factors in post-menopausal women [24].

There is increased mortality after hip fracture [4]. Patients who sustained CVA after a hip fracture had increased risk of mortality relative to patients without CVA. The combined effect of CVA and hip fracture decreases survival and attempts to reduce the incidence of CVA should be made. Hip fracture itself is a negative prognostic factor for CVA as shown by this study. There was a 17.5% incidence of CVA in our cohort, which is appreciably greater than the rate of CVA in the general population (reported as 6.1 and 5.2% in males and females respectively between the ages of 60–79) [10, 25].

Forty-one (1.9%) of all patients in this study sustained CVA during the first year. This is different from previously reported data that describe between 3.9 and 4.1% of patients developed CVA in the first year postoperatively [9, 10]. This may be partially explained by short time from admission to surgery and the routine use of low-molecular-weight heparin for venous thromboembolism prophylaxis (Enoxaparin 40 mg IM) for 35 days from the time of admission in this cohort.

### Limitations

Limitations of this study include the lack of a non-hip fracture control group. Patients did not receive uniform surgical intervention. Fracture type may also impact the development of CVA. Pre-fracture mobility and activities of daily living data were not recorded. Patient compliance with enoxaparin usage and rehabilitation protocol was not documented. Co-morbidities were queried from medical recorders and there may be an underreporting of comorbidities.

## Conclusion

Hypertension, diabetes mellitus, and atrial fibrillation are significant risk factors for the occurrence of CVA after hip fracture. The majority of CVAs occur between the first and fifth year postoperatively. CVA is a negative prognostic factor for postoperative survival. Therefore, emphasis should be given in this particular group of patients in the first 5 years after hip fracture in order to try to reduce the mortality.

## Abbreviations

AF: Atrial fibrillation
CHF: Congestive heart failure
CRF: Chronic renal failure
CVA: Cerebrovascular accident
DM: Diabetes mellitus
DVT: Deep vein thrombosis
FWB: Full weight bearing
HCL: Hypercholesterolemia
HTN: Hypertension
IHD: Ischemic heart disease
NWB: Non-weight bearing
PE: Pulmonary embolism
PWB: Partial weight bearing
WBAT: Weight bearing as tolerated
